# Diagnosing inflammation and infection in the urinary system via proteomics

**DOI:** 10.1186/s12967-015-0475-3

**Published:** 2015-04-08

**Authors:** Yanbao Yu, Patricia Sikorski, Cynthia Bowman-Gholston, Nicolas Cacciabeve, Karen E Nelson, Rembert Pieper

**Affiliations:** The J. Craig Venter Institute, 9704 Medical Center Drive, Rockville, MD 20850 USA; Quest Diagnostics at Shady Grove Adventist Hospital, 9901 Medical Center Drive, Rockville, MD 20850 USA; Advanced Pathology Associates LLC at Shady Grove Adventist Hospital, 9901 Medical Center Drive, Rockville, MD 20850 USA; Current affiliation: Department of Microbiology and Immunology, Georgetown University, 37th and O Streets, NW, Washington, D.C., 20057 USA

**Keywords:** Urinary tract infection, Innate immune response, Inflammation, Neutrophil infiltration, Urine diagnostics, Proteomics, Metaproteomics, *Gardnerella*, *Klebsiella*, Uropathogen

## Abstract

**Background:**

Current methodology for the diagnosis of diseases in the urinary system includes patient symptomology, urine analysis and urine culture. Asymptomatic bacteriuria from urethral colonization or indwelling catheters, sample contamination from perineal or vaginal sources, and non-infectious inflammatory conditions can mimic UTIs, leading to uncertainty on medical treatment decisions.

**Methods:**

Innovative shotgun metaproteomic methods were used to analyze urine sediments from 120 patients also subjected to conventional urinalysis for various clinical reasons including suspected UTIs. The proteomic data were simultaneously searched for the presence of microbial agents, inflammation, immune responses against pathogens, and evidence of urothelial tissue injury. Hierarchical clustering analysis was performed to identify host protein patterns discerning UTI from urethral colonization and vaginal contamination of urine samples.

**Results:**

Organisms causing more than 98% of all UTIs and commensal microbes of the urogenital and perineal area were identified from 76 urine sediments with detection sensitivities estimated to be similar to urine culture. Proteomic data permitted a thorough evaluation of inflammatory and antimicrobial immune responses. Hierarchical clustering of the data revealed that high abundances of proteins from activated neutrophils were associated with pathogens in most cases, and correlated well with leukocyte esterase activities and leukocyte counts via microscopy. Proteomic data also allowed assessments of urothelial injury, by quantifying proteins highly expressed in red blood cells and contributing to the acute phase response. *Lactobacillus* and *Gardnerella vaginalis* were frequently identified suggesting urethral colonization and/or vaginal contamination of urine.

**Conclusions:**

A metaproteomic approach of interest for routine urine clinical diagnostics is presented. As compared to urinalysis and urine culture methods, the data are derived from a single experiment for a given sample and provide additional insights into presence or absence of inflammatory responses and vaginal contamination of urine specimens.

**Electronic supplementary material:**

The online version of this article (doi:10.1186/s12967-015-0475-3) contains supplementary material, which is available to authorized users.

## Background

Urologic diseases are among the most common diseases worldwide. Specific and informative methods for their diagnosis are important to benefit patients and potentially reduce overuse of antibiotic drugs. An estimate of UTI cases occurring globally per year is 150 million [1]. Annual expenditures for Medicare beneficiaries in the USA (65 years and older) for non-cancerous diseases of the urinary tract were estimated to be 8.5 billion in 2009 U.S. dollars including treatment in and outside of hospitals. Sixty percent of these costs pertain to the treatment of UTIs [2]. Women are at least five times more likely to contract a UTI at least once during their lifetimes than men due to anatomical differences [2] because bacteria are more easily able to enter and colonize the female urethra. Catheter-associated UTIs (CAUTI), a growing public health concern, are often hospital-acquired and not reimbursed by medical insurance companies as they are considered to be preventable [3]. CAUTI-associated bacteriuria leads to higher incidence of pyelonephritis and urosepsis [4], and is often asymptomatic in patients resulting in uncertainty about the necessity to treat with antibiotic drugs [3]. Approximately 25 million urinary catheters are used in the USA alone, and 15% of the admitted patients in acute care hospitals are catheterized at least once [5]. Uropathogenic *Escherichia coli* (UPEC) is the most common causative agent of UTI, while others are *Enterococcus faecium*, *Staphylococcus aureus*, *Klebsiella pneumoniae*, *Pseudomonas aeruginosa* and *Enterobacter* spp. These bacteria belong to the “ESKAPE” group of pathogens feared to become untreatable in the near future due to multiple-drug resistance and/or tolerance [6].

Non-infectious diseases of the urinary system such as interstitial cystitis, chronic prostatitis, tubulointerstitial nephritis, and kidney stone formation can mimic UTIs. Urine analysis (UA) tests for proteinuria and hematuria and microscopic evaluation of renal cell and erythrocyte casts can be useful for the diagnosis of kidney diseases including nephrolithiasis [7,8], but are not sufficiently specific. Potassium sensitivity testing and cystoscopy are diagnostic methods used for patients with symptoms of chronic cystitis and prostatitis [9]. Tissue biopsies provide more accurate diagnoses for diseases of the urinary system, but are invasive and expensive. Unlike soluble urine, urinary sediments have rarely been considered as a source of biomarkers indicative of non-infectious disorders in the urinary system.

Overuse of antibiotic drugs for suspected UTIs is common, and error rates of UTI diagnosis based on clinical criteria are estimated to be as high as 33% [10]. Testing for UTI is currently a multi-stage process and requires up to 48 h from the time of clean-catch urine collection to the identification of the causative microbial agent(s). Dipstick UA tests estimate the quantities of protein, blood, nitrite and leukocyte esterase (LE) in a patient’s urine sample. Typical, but not universally used positive scores are ≥1, ≥1.5, and ≥2, respectively [10]. Microscopic analysis of a urine sediment permits semi-quantitative measurements of leucocytes and epithelial cells shed into urine. The leukocyte count and LE activity score are the principal measures of inflammation. Urine culture (UC) uses selective media to count colony forming units (CFU) followed by Gram stains and additional chemical assays to identify the types of bacteria present. Based upon patient symptoms, signs of disease, the types of bacteria identified, CFU numbers and the type of urine collection (clean catch versus catheter and suprapubic aspiration), clinical assessments are made. However, it is sometimes difficult to distinguish UTIs from asymptomatic bacteriuria (ASB) and contamination by microbes from the perineal and genital areas, which necessitate different treatment for each. The current diagnostic pathway used in the clinical setting may fail to detect pathogens due to recent antibiotic usage (false negative) and detect bacterial colonization or urine sample contamination from inadequate collection (false positive). Methods that improve diagnostic precision and are better able to distinguish UTIs from other medical conditions with similar symptoms are desirable.

Technological advances such as matrix-assisted laser desorption ionization time of flight (MALDI-TOF) mass spectrometry (MS) have begun to impact strategies for bacterial identification in the clinical setting. While MALDI-TOF is fast, sensitive and specific when performed on isolated bacterial colonies, it is less specific when more complex samples such as urinary sediments are examined [11-13]. MALDI-TOF also requires the acquisition of customized instruments and software tools, thus limiting its use to larger clinical laboratories. The method neither characterizes immune responses to infection nor urogenital inflammation not caused by microbial agents. The utility of new UTI diagnostic tests targeting specific bacterial genes or proteins and measuring components of the human innate immune system were mentioned as early as 2001 [1]. Inflammatory responses against infectious agents in the urinary tract have been characterized [14,15] and implicate the binding of pathogen recognition receptors to bacterial lipopolysaccharides and other cell envelope structures as well as the release of inflammatory chemokines. Chemokines initiate leukocyte extravasation and their migration to the sites of infection. Neutrophils are the main cell type involved in phagocytosis and killing of the invading uropathogens [14,15]. The complement system is implicated in bacterial opsonization in the urinary tract. Mediated by the binding of complement component C3b to urothelial cell receptors, C3b-opsonized UPEC cells are internalized by the epithelial cells and survive [16], a pathway that also appears to enable the formation of quiescent intracellular bacterial communities [17].

We developed the concepts of metaproteomic analysis using urine samples in a study on bacteriuria in spinal cord-injured patients [18]. The term is defined as the simultaneous analysis of proteins derived from several organisms, including pathogen and host if applicable, and is implemented by database searches matching mass spectral data to protein sequences representing organisms likely present in the analyzed sample. We analyzed 120 urine samples obtained from a clinical pathology laboratory that previously examined the samples with standard urinalysis tests. To determine the diagnostic value of the proteomic method, we compared subsets of the proteomic data with equivalent results from UA and UC tests. This comparison pertains to evidence of bacterial pathogens, leukocyte infiltration in the urinary tract, tissue injury and hematuria, and vaginal contamination of urine.

## Methods

### Study design

Metaproteomic analysis allows simultaneous identification (ID) of proteins from several organisms. In this study, it is a host organism and the microbes able to colonize the human urogenital tracts. Mass spectrometry-based proteomic analyses rely on database searches. The database used here included the protein sequences from 21 microbial genomes (species) including those responsible for more than 98% of all UTIs [19] and urogenital commensal organisms. We analyzed urine sediments in which both microbial cells and molecular and cellular effectors of the human immune response are enriched. We compared the proteomic data pertaining to UTIs, urethral colonization by microbes, and sample contamination from vaginal sources with microscopic and dipstick UA test as well as UC test results. Surveying a relatively large number of patients (120), we were able to evaluate methods to quantify the overall intensity of immune responses and tissue injury from proteomic data and to conduct hierarchical clustering analyses to group patients into distinct categories related to immune response characteristics and potential vaginal contamination.

### Human urine specimens

The urine specimens were from the Pathology and Clinical Microbiology Laboratory of Shady Grove Adventist Hospital (SGAH), Rockville, MD. Standard UA tests ordered by SGAH physicians were performed. Collection tubes and the respective UA/UC records were de-identified. To be selected for proteomic analysis, urine specimens had to meet two of three score thresholds: nitrite concentration ≥ 1+; LE activity ≥ 1+ (each measured by dipstick tests); bacteria ≥ 1+ in urine sediments (assessed by microscopy). Urine samples (5 to 30 ml) were stored at 4°C for up to 6 h and centrifuged at 3,000 × g for 15 minutes at 10°C. Urinary pellet (UP) samples were washed twice with a *circa* 10-fold volume of PBS and frozen at −80°C until further use.

### Urinalysis and urine culture methods

Urinalysis testing was performed using the Iris Diagnostics AUTION MAX AX-4280 analyzer. Test strips containing chemically impregnated pads are dipped into urine samples and then placed on the analyzer. For the detection of nitrite, the Griess reaction is performed with the addition of Sulfaniliamide and N-1Naphthylethylenediamine dihydrochloride [20]. For the detection of leucocyte esterase, 3-(N-Toluenesulfonyl-L-alanyloxy)indole is used for colorimetric measurements [21]. Urine samples were cultured by using a standard calibrated loop to deliver 0.001 ml of urine to inoculate and cross-streak Trypticase Soy Agar with 5% sheep blood and MacConkey agar plates. These were incubated at 35°C in air atmosphere for a minimum of 18 h. The number of bacterial colonies represented 1,000 CFUs per ml. Bacterial isolates were identified using the BioMerieux Vitek 2 instrument (BioMerieux, Inc. Durham, NC).

### Preparation of urinary pellet samples for proteomic analysis

The UP samples were processed as previously described [22]. Briefly, re-suspension in a lysis solution containing 8 M Urea, 1% SDS, 5 mM Na-EDTA, and 50 mM DTT was followed by sonication at amplitude 6 (Misonix 3000, Ultrasonic Cell Disruptor) in six 45 s on/off cycles while cooling the lysates in an ice-water bath. After a 10 min centrifugation step at 16,000 × g, the soluble lysate fraction was collected, and the protein amount was estimated from Coomassie Blue G250-stained gel bands in SDS-PAGE gels. The filter-aided sample preparation method called FASP [23] was employed with minor modifications [22] using Vivacon filter devices with a 30 kDa MWCO (Sartorius AG, Germany) including two consecutive tryptic digestion steps. Peptide mixtures were eluted and lyophilized prior to clean-up with the Stage-Tip method [24].

### Nano LC-MS/MS method for proteomic analysis

The nano LC-MS/MS method was adapted from a previous publication with minor modifications [22]. An Ultimate 3000-nano LC and a Q Exactive mass spectrometer system coupled via a FLEX nano-electrospray ion source (all components from Thermo Scientific, West Palm Beach, FL) were used. Peptide samples were separated on a PicoFrit analytical column (75 μm × 10 cm, 5 μm BetaBasic C_18_, 150 Å, New Objective, MA) at a flow rate of 300 nl/min. A 130 min LC gradient was applied. The gradient started with 98% solvent A (0.1% formic acid in water), and increased to 35% solvent B (0.1% formic acid in acetonitrile) over 110 min, followed by a steeper gradient to 80% solvent B over 15 min. Eluting peptides were sprayed at a voltage of 2.0 kV and acquired in a MS data-dependent mode using XCalibur software (version 2.2, Thermo Scientific). Survey scans were acquired at a resolution of 70,000 over a mass range of m/z 250 to m/z 1,800 with an automatic gain control (AGC) target of 10^6^. For each cycle, the ten most intense ions were subjected to fragmentation by high energy collisional dissociation with normalized collision energy of 27%. Peptide ion fragments from the MS/MS scans were acquired at a resolution of 17,500 with an AGC target of 5 × 10^4^. Dynamic exclusion was enabled, with MS/MS ion scans repeated once and then excluded from further analysis for 20 s. The unassigned ions and those with a charge of +1 were rejected from further MS/MS analysis. All LC-MS/MS experiments were run in duplicates. To minimize instrumental variation, the system was regularly calibrated with positive ion calibration solutions (Pierce, Rockford, IL) until 0.1 ppm mass accuracy was achieved.

### Proteomic database searches and methods for quantification

Raw spectral files acquired by the MS system were processed using the Proteome Discoverer platform (version 1.4, Thermo Scientific) and the MaxQuant software suite [25]. A workflow using the algorithm Sequest HT was employed to identify proteins. We modified the complete human UniProtKB database (Release 2013_6; 88,295 human sequences) by using a 75% sequence identity threshold with an application in the CD-HIT suite [26] to reduce protein redundancies. This database contained 27,151 human protein sequences and was combined with protein sequences from 21 microbial genomes of species colonizing the human urinary tract (Additional file 1: Table A1). The final database consisted of 97,919 protein sequences. The false discovery rates (FDR) were determined by searching reversed versions of the database [27]. Only proteins and peptides with a 1% FDR were accepted. For protein quantification of the datasets, the MaxQuant software suite (version 1.4.2) which integrated MS^1^ peak areas from high-resolution MS was used [28]. Most of the default settings were accepted, and data were processed using the intensity-based absolute quantification (iBAQ) and total protein analysis (TPA) methods [29]. For TPA analysis, each protein i’s normalized spectral protein intensity LFQ_i_ was divided by the sum of all LFQ intensities of the measured proteome; each LFQ_i_/ΣLFQ quotient was divided by the number of theoretical tryptic peptides per protein. The number of theoretically observable peptides is calculated by *in silico* digestion of protein sequences including fully tryptic peptides with a length of six to thirty amino acids. TPA data reflected the averaged protein quantities from duplicate LC-MS/MS analyses. Only the ‘leading sequence’ member for each protein group was included in the computations; protein groups are defined according to parsimony principles of peptide assignments to proteins of origin. Identification of at least two unique peptides per protein group was required for quantification of the data. A scoring scheme based on summed iBAQ quantities for the protein groups was developed to allow a direct comparison of UA results with the proteomic data. Proteins identified to quantitatively assess neutrophil activation and degranulation (NAD score), complement system activation and coagulation (CAC score), red blood cell content (ERY score), and vaginal epithelial contamination of urine (VCO score) are listed in detail in (Additional file 2: Table A2).

### Statistics

The Wilcoxon rank sum test is a nonparametric test of the null hypothesis that two populations are the same against an alternative hypothesis and is more efficient on non-normal distributions than t-tests. The test was used to assess statistical differences of VCO scores comparing different sets of UP sample clusters. Hierarchical clustering analyses (HCL) of UP samples (or human subjects) and proteins were performed using an application in the software Multiple Experiment Viewer (MeV) [30]. To perform HCL, we imported the quantitative data matrix derived from the TPA method for 110 UP samples into MeV following the selection of human protein entries only, and transformed the data logarithmically. Missing values were replaced by −30 to allow including them as data points into the HCL analysis. The TPA values ranged from −28 to −5 in abundance. We used the Pearson correlation metric for HCL. The clustering parameters were defined as followed: (1) selection of gene (protein) and UP sample trees; (2) optimization for gene tree order to analyze clusters of proteins or UP sample tree order to analyze clusters of human subjects; (3) the absolute distance metric and complete linkage clustering were applied in each case.

### Study approval

Patients were not selected for this study based on specific diagnoses or medical histories. Specimens were de-identified and considered medical waste, and the study was exempted from review by the internal review boards of Shady Grove Adventist Hospital (SGAH) and the J. Craig Venter Institute (JCVI).

## Results

### Sample sources to evaluate urinary proteomic data for urine diagnostic purposes

The collection of 120 urine samples profiled in this study was not limited to the diagnosis, assessment of progression or treatment of a specific disease. Urinalysis (UA) of the samples was ordered by attending physicians for various reasons including acute injury, vaginal bleeding, dizziness/nausea, hyperlipidemia, Type II diabetes and associated complications, abdominal pain/nausea, unspecified hypertension, bladder hypertension, idiopathic polyuria, and suspected UTI. Since UTI is a highly prevalent infectious disease linked to some of the aforementioned clinical symptoms (e.g., abdominal pain/nausea) and risk factors (e.g., diabetes), we expected frequent diagnosis of bacteriuria or UTI from these specimens. Proteomic analyses were limited to specimens where urinalysis reports provided tentative support for bacterially caused UTIs (see Methods). We did not analyze urine specimens in cases where the specific diagnosis of asymptomatic bacteriuria was made available. Extensive UA data were obtained for all of the 120 samples. This included dipstick tests, microscopic examination of urinary sediments for various cell types and mucus, and - in 46% of the cases - urine culture (UC) data. Data on urine appearance such as turbidity, color and urine pellet color and volume were also reviewed. Urinalysis results allowed a comprehensive comparison of the conventional methods to diagnose diseases of the urinary tract with data from metaproteomic surveys (Additional file 3: Table A3).

### Neutrophils, the dominant effectors of innate immune responses in the urinary tract, account for high levels of inflammation in many samples

**Figure 1:**
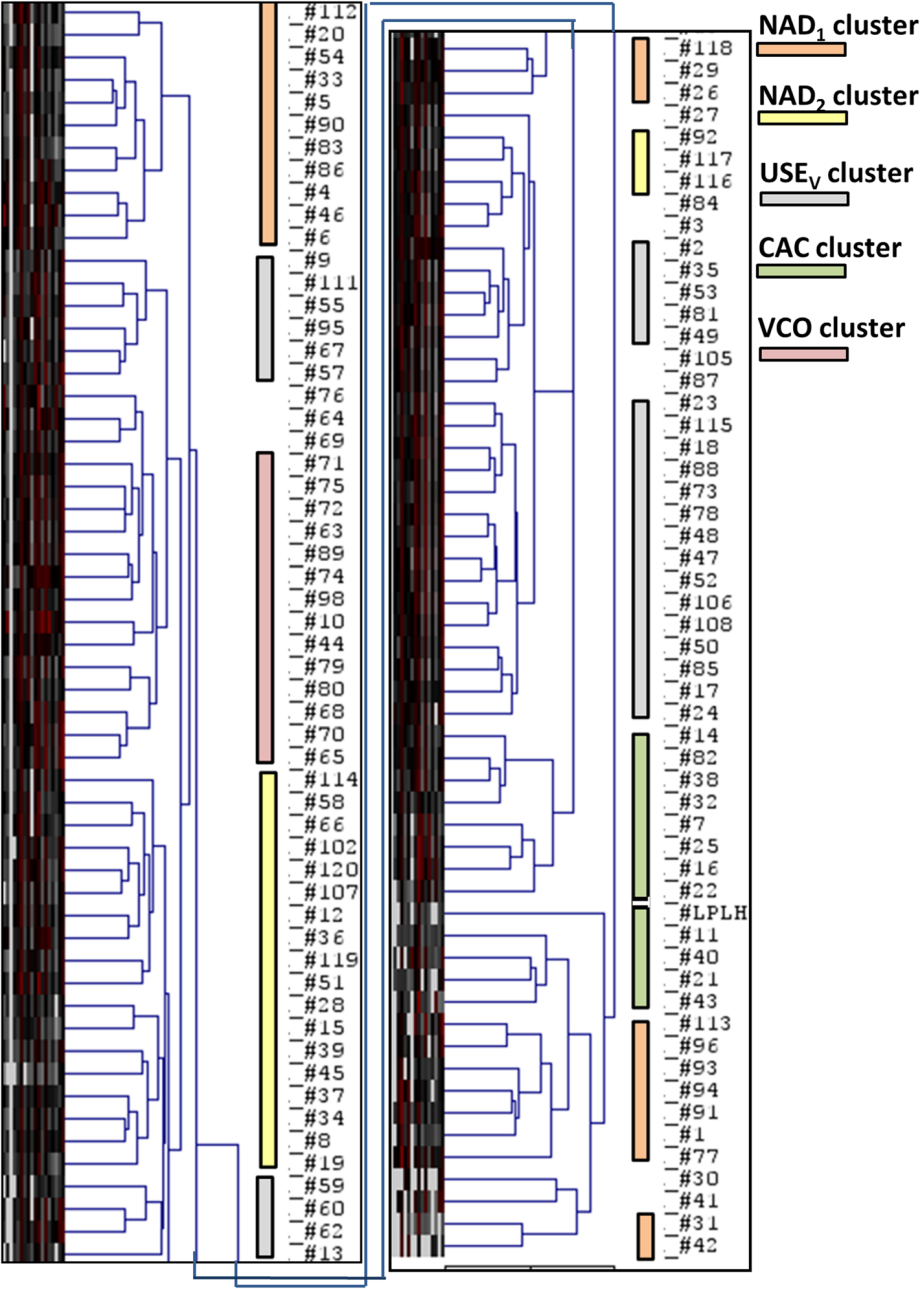
**Hierarchical clustering analysis of urinary pellet proteomic profiles for 110 samples.** Hierarchical clustering was performed on quantitative human protein datasets using the TPA method in the MaxQuant software. The datasets were subjected to the Pearson correlation analysis with sample leaf order-optimization and complete linkage clustering using the software tool MeV [30]. We eliminated the protein abundance heat map from the displayed hierarchical tree of the UP samples. The bottom of the panel on the left side connects to the top of the panel on the right side as it pertains to tree linkages. The sample cluster names, shown on the far right of the graphic with their acronyms, are discussed in detail in the text. Colored bars indicate the type, size, and position of each sample cluster in the tree.

Proteomic data yielded strong evidence for the important role of neutrophils as effectors and messengers of inflammation in the urinary tract. Neutrophils release antimicrobial and inflammatory molecules from secretory granules they produce and kill invading pathogens in phagolysosomes after their phagocytosis. A hierarchical clustering analysis optimized for sample leaf order (HCL_SO_) identified four sample clusters with human protein abundance profiles dominated by neutrophils (23 of 111 cases; NAD_1_ clusters in Figure 1) and two clusters with neutrophil-specific protein quantities comparable to those associated with the cytoskeleton (25 cases; NAD_2_ clusters in Figure 1). Cytoskeletal proteins are highly expressed in epithelial cells lining the urogenital tract and make up the majority of the urinary sediment proteome in the absence of pathophysiological conditions in the urinary tract. Thirty-five of the 48 NAD cluster profiles were positive for IDs of a uropathogen including *G. vaginalis*, indicative of the fact that a dominant reason for inflammation in the respective patients was bacteriuria and an immune response towards the invading microbes. The reason for the distances among the NAD clusters in the linkage tree was the considerable variation in the number of identified human proteins ranging from 200 to 1,500 IDs per sample.

### Neutrophil protein abundances derived from proteomic data correlate well with LE activities and leukocyte counts

**Figure 2:**
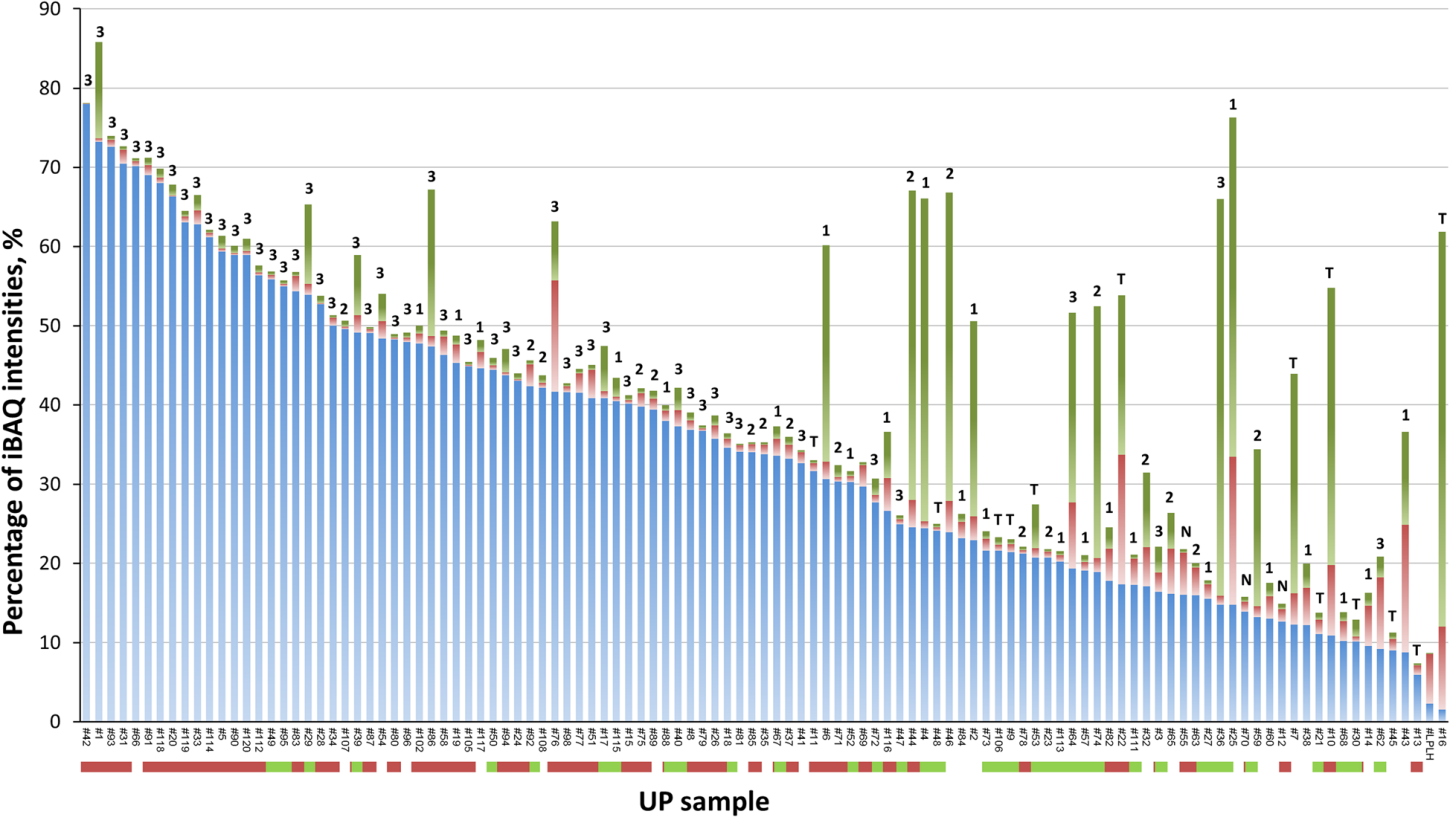
**Protein profiles showing quantitative contributions of neutrophils, the complement system, and erythrocytes to the total proteome of urinary pellet samples.** The graph displays the summed protein abundances, relative to the total proteome, for three biological protein categories. The x-axis lists the identifiers of the UP samples associated with 110 human subjects. The three categories represent proteins produced by activated neutrophils (BLUE), proteins highly expressed in erythrocytes and released upon vascular injury (GREEN), and proteins associated with complement system activity and coagulation (RED). The method used for quantification of all proteins is the iBAQ method in the MaxQuant software tool. The order of samples is based on neutrophil protein abundance, decreasing from left to right. To allow direct comparisons for the assessment of inflammation, the score for the LE assay was included in the graphic above each bar representing a sample. Underneath the x-axis, an additional bar depicts which samples were associated with the ID of a pathogen causing UTI (ORANGE color segments), commensal bacteria (GREEN color segments) or lack of bacterial IDs (no color).

**Figure 3:**
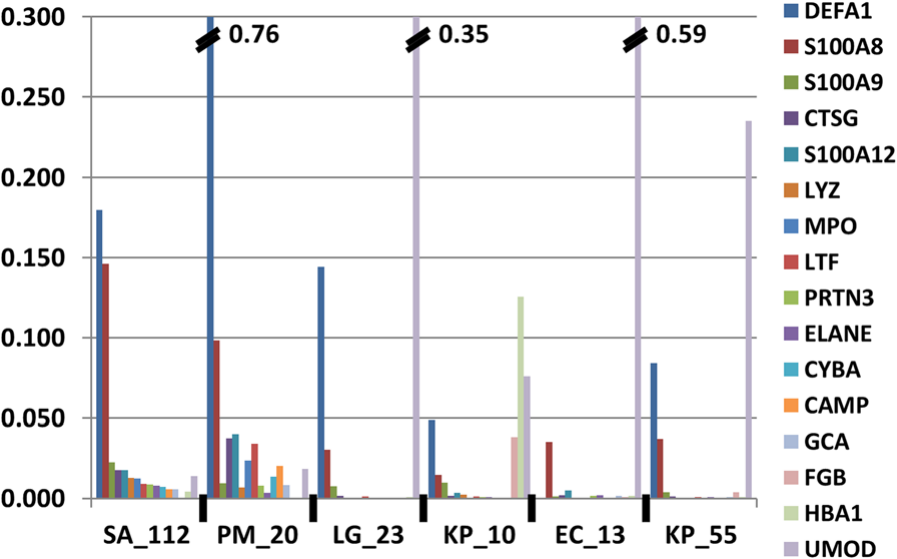
**Abundance of selected neutrophil proteins in UP samples.** Thirteen proteins listed in the legend on the far right are neutrophil granule proteins. Three other proteins are fibrinogen-β (FGB; implicated in coagulation), hemoglobin α-subunit (HBA1; indicative of vascular injury), and uromodulin (UMOD; abundant in urine of healthy donors). The samples SA_112 and PM_20 represented UTIs caused by *S. aureus* (SA) and *P. mirabilis* (PM), respectively. The profile of LG_23 (*Lactobacillus*) indicated the lack of inflammation, and represented urethral colonization, possibly also minor vaginal contamination of the urine sample. KP_10 (*K. pneumoniae*) appeared to represent vaginal infection as vaginal bleeding was clinically diagnosed for the patient. The protein profiles of EC_13 (*UPEC*) and KP_55 suggested the near-absence of inflammation, and therefore most likely urethral colonization. Proteins were quantified using the iBAQ method, in each case divided by the summed iBAQ values for the entire UP proteome.

A key motivation for this study was to determine how proteomic data derived from urine sediments compared to conventional assays to quantify levels of inflammation in urine samples. We determined the quantities of 35 proteins known to be highly expressed in activated neutrophils (Additional file 2: Table A2) relative to total protein abundance for each UP sample, as shown in blue bar segments of the plot displayed in Figure 2. Not unexpectedly, 85% percent of cases belonging to the aforementioned NAD_1_ clusters were in the section of the graph with greater than 30% neutrophil protein content (on the left side). The 35 proteins included five functional groups: the calcium-binding S100 family proteins S100-A8, S100-A9, and S100-A12 which contribute 40-50% of the total cytosolic protein content in neutrophils; proteins released during inflammation from neutrophil granules, including myeloperoxidase (MPO), cathepsin G (CTSG), defensin-1 (DEFA1), elastase (ELANE), lysosome (LYZ), lactotransferrin (LTF), and cathelicidin (CAMP) [31]; proteins implicated in the formation, trafficking, and fusion of granules with phagolysosomes, including grancalcin (GCA), plastin-2 (LCP1), annexin A3 (ANXA3), and tetraspanin (CD63 antigen); proteins influencing neutrophil migration in the milieu of a reorganizing extracellular matrix, such as integrin αM/β2, gelatinase (MMP9), and neutrophil collagenase (MMP8); and NADPH oxidase, an enzyme with multiple subunits including cytochrome b-245 (CYBA, Figure 3) located in the membrane of phagolysosomes of phagocytic cells and responsible for the oxidative burst that directly kills pathogens. Many of these proteins, especially defensin-1, were highly abundant in samples with evidence of UTIs (e.g., SA_112 and PM_20, Figure 3). The bacterial pathogens in the two cases were *S. aureus* (SA) and *P. mirabilis* (PM) and, not unexpectedly, were located on the left side in the plot of Figure 2 and adjacent to each other in an NAD_1_ cluster (Figure 1). We acknowledge that this data reflects approximate quantities of neutrophils considering the fact that proteins such as defensin-1, LTF, S100-A8, and S100-A9 are also released into the urinary tract by urothelial cells. However, hierarchical clustering analysis optimized for protein leaf order (HCL_PO_) showed that these proteins clustered with each other, supporting the notion of a dominant role of neutrophils in their production (Additional file 4: Figure A1). Eosinophil peroxidase (EPX) and eosinophil cationic protein (ECP) both of which are also effectors of the response towards pathogens were an order of magnitude less abundant than neutrophil-derived proteins, thus not supporting a major role of eosinophils in the inflammatory response. The macrophage-specific migration inhibitory factor (MIF) was present in even lower quantities, suggesting the near-absence of macrophages as participants in acute immune responses following pathogen invasion in the urinary tract.

### NAD score

The representation of neutrophil protein quantities in the plot of Figure 2 is derived from the NAD (neutrophil activation and degranulation) scores also provided in (Additional file 3: Table A3). Figure 2 includes LE scores ranging from negative (N) to trace (T), 1, 2, and 3 for each sample. Overall, a strong correlation of neutrophil protein abundances and LE scores is observed. The LE score measures leukocyte esterase activity, likely representing primarily elastase (ELANE) and myeloblastin (PRTN3), two neutrophil proteases quantified for eight samples in Figure 3. All profiles on the left side of the graph (Figure 2) and fifty-three of the 60 samples with more than 30% (iBAQ) neutrophil protein content had LE scores of 2 or 3. Twenty-nine of the 40 samples with less than 23% (iBAQ) neutrophil protein content had LE scores ranging from negative to 1. In only four of eleven cases where the LE score was ≥ 2, but the neutrophil protein content was relatively low, the microscopic leukocyte count was greater than 11 cells per high power field (HPF), a threshold used to define pyuria. Overall, there was slightly less agreement in the comparison of leukocyte counts setting the threshold at 11 cells/HPF with more than 30% (iBAQ) neutrophil protein content: 14 of the 60 cases had counts ≤ 10 (Additional file 3: Table A3). In summary, assessing neutrophil content in urine sediments via proteomics appears to be at least as accurate as the LE assay to diagnose inflammation in the urinary tract. It measures the sum of abundances of 35 proteins enriched in neutrophils and may be less susceptible to false positive results compared to the LE assay.

### Abundance of erythrocyte protein content serves as diagnostic indicator of vascular injury

Vascular injury in the urinary tract, typically associated with inflammation, is assessed with dipstick tests for hemoglobin and microscopic counting of red blood cells in conventional urinalysis. Considering the high enrichment of distinct proteins in erythrocytes, we were able to develop a proteomic approach equivalent to conventional tests for hematuria. Summed abundances of 32 red blood cell proteins, including hemoglobin subunits, band 3 anion transport protein, band 7 integral membrane protein, and carbonic anhydrase-1, relative to total protein content in each UP sample were determined, shown by the green bar segments of the plot displayed in Figure 2. These protein quantities are listed as ERY scores in (Additional file 3: Table A3) for each sample. There was no evidence of a good correlation of either NAD scores or LE assay results with ERY scores, suggesting that, even in the cases of detection of a pathogen (shown by the coloration of the horizontal bar at the bottom of the plot in Figure 2), neutrophil infiltration of the urinary tract does not always entail significant hematuria and tissue injury. Setting the thresholds for hematuria at 2+ for the dipstick test and 4.5% for the ERY score, there was agreement among 81% of all cases. For the 21 cases where scores disagreed, microscopic red blood cell counts were assessed. Using a count greater than 10 cells per high power field (HPF) as evidence of hematuria, we found that in two thirds of the cases the microscopic analysis was in agreement with proteomic data. We conclude that the proteomic ERY scores provide a good quantitative estimate of hematuria in urine.

### Urine samples enriched in proteins implicated in complement activities and coagulation

We observed that the HCL_PO_ analysis applied to all urinary proteomic profiles clustered 21 proteins with functional roles in coagulation pathways and/or the complement system (Additional file 4: Figure A1). The rationale for measuring protein abundances pertaining to these inflammatory pathways jointly was also based on reports of extensive functional interactions [32]. We identified 42 proteins associated with complement system activities and coagulation (CAC) whose summed abundances are included as the CAC score for each UP sample in (Additional file 3: Table A3). The complement system contributes to the acute phase response and innate immunity, and distinct components are pro- or anti-inflammatory. A central component is complement component C3. C3 matures into the opsonin C3b and the anaphylatoxin C3a and is secreted into blood plasma and the urinary tract following production in renal tubular cells [33]. C3 plays a role in upper urinary tract infection [34] and the uptake into and quiescence of UPEC in uroepithelial cells possibly associated with the clinical problem of recurrent UTIs [17]. Even though the quantities of proteins linked to complement activities and coagulation were not as high neutrophil proteins, the HCL_SO_ analysis generated two sample clusters, adjacent in the tree and with a total number of 12 samples, characterized by relatively high abundance of such proteins (the CAC clusters in Figure 1). CAC clusters revealed a low number of cases with an ID for a pathogen (3 of 12). CAC scores were plotted in Figure 2 depicted by the red bar segments of each sample (column). High overall quantities of CAC proteins did not correlate well with high quantities of neutrophil proteins suggesting that inflammatory activities mediated by the complement system (e.g., C3 and C4) and coagulation (e.g., fibrinogen) may be separately regulated upon pathogen invasion or other stresses the urinary tract was exposed to in patients. High CAC and ERY protein quantities were more frequently observed to occur in tandem. Many coagulation and complement proteins are indeed abundant in blood plasma. This body fluid leaks into the urinary tract lumen upon vascular injury. Urinalysis tests equivalent to the measurement of CAC scores are rarely used in clinical laboratories.

### Precipitation of uric acid salts and associated urine sediment proteomic profiles

**Figure 4:**
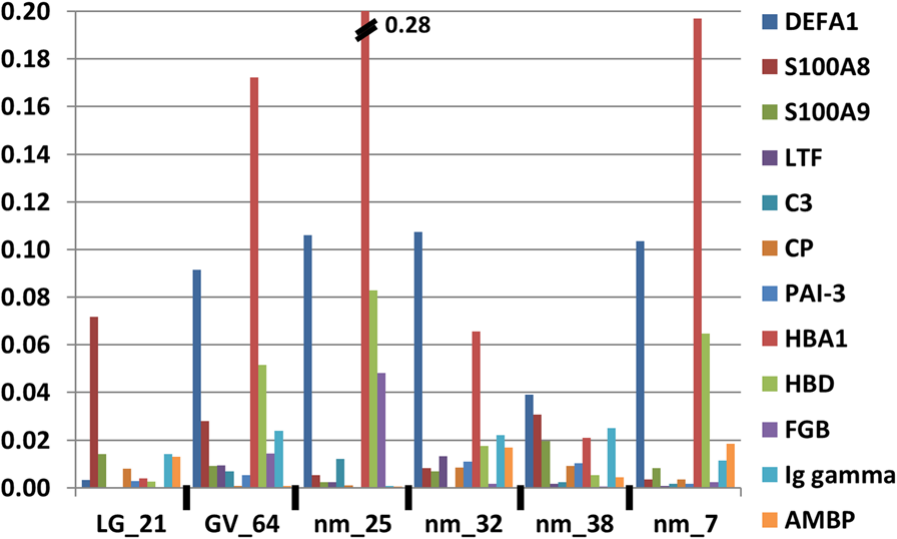
**Abundance of selected proteins in samples with evidence of salt precipitation in urine.** UP samples beginning with nm_ (no microbes) did not show evidence of bacterial colonization, but the urine sediments had a visual appearance suggesting uric acid salt precipitates and were present in two CAC clusters. LG_21 (*Lactobacillus*) represented the absence of inflammation. The patient associated with sample GV_64 (*G. vaginalis*) was diagnosed with kidney stones. In addition to those proteins described in the legend of Figure 3, others are the complement component C3 (C3), ceruloplasmin (CP), plasminogen activator inhibitor-3 (PAI-3), AMBP (bikunin), hemoglobin δ-subunit (HBD) and immunoglobulin γ-chain (Ig gamma). All of these proteins are implicated in the acute phase response, which is typically initiated by tissue injury and pathogen invasion. The sample profiles show high variability of protein quantities, although distinct acute phase proteins were increased compared to the control LG_21 in some of the samples.

Visual inspection of the 12 UP samples in the CAC clusters revealed that nine urine samples were highly turbid, and ten urine pellets relatively large with a pink-to-light brown coloration. These features have been linked to high saturation levels of uric acid and precipitation of uric acid salts, especially at a pH below 6, in urine. Precipitation of uric acid can be a precursor state of urinary stone formation [35,36]. It is reasonable to assume that the samples’ cloudy appearances contributed to mistakenly identifying the precipitates as bacteria during microscopy. Only one clinical record was available reporting the occurrence of kidney stones (GV_64). Although the proteomic profile for this patient was not part of the CAC cluster, the visual features of the sample also stated turbidity and a pink-to light brown urine pellet color. We assume that proteins relatively abundant in the soluble fraction of urine bind to the salt precipitates and therefore contribute to distinct protein abundance patterns in the respective UP samples. Indeed, proteins generally soluble in urine and normally of low abundance in urinary pellets were increased in abundance in some CAC cluster samples relative to a control without evidence of UTI and salt precipitates (LG_21). Examples of such proteins are IgG γ-chain, AMBP (bikunin), and fibrinogen γ-chain, as shown in Figure 4. The proteins defensin-1 and the subunits HBA1 and HBD of hemoglobin were most strongly increased in abundance compared to data for LG_21. Although most proteins displayed in Figure 4 are known to be increased in urine and plasma as a consequence of local injury and contribute to the acute phase response, this data showed high quantitative variability. Pathological significance, specifically injury in the urinary tract, cannot be inferred from this data. Proteomic profiles were recently reported for the urinary stone matrix [37]. Among the most frequently observed proteins in the stone matrix were IgG heavy chains, fibrinogen subunits, S100-A8, lysozyme C, and LTF, proteins also shown in the plot of Figure 4. Further investigations are needed to evaluate the value of proteomic analysis to identify biomarkers from urine samples containing urine salt precipitates, e.g. to assess the risk of kidney stone formation.

### Urethral colonization by commensal bacteria not eliciting host immune responses

Highly parallel DNA sequencing technologies have revealed that urine is not completely sterile, and the notion of a urinary microbiome has evolved as an interesting research topic [38]. It is likely that external parts of the urethra, especially in women, are colonized with bacteria from perineal and vaginal sources. It is also evident that suboptimal collection of clean-catch urine from female patients can result in the contamination of urine with proteins and commensal bacteria from the vaginal cavity. Data presented here can be explained by, but do not distinguish the two aforementioned scenarios. Approximately 25% of the urinary proteome profiles obtained in this study were enriched for proteins secreted into urine in a physiologically normal milieu (e.g., uromodulin and cytokeratins) or abundantly produced by epithelial cells shed by mucosal surfaces lining the urinary and vaginal tracts. The HCL_SO_ analysis identified four clusters (Figure 1), one large cluster with 15 UP samples, derived from female patients with only two exceptions. The profiles revealed high abundance of cytoskeletal proteins (e.g., α-actins and annexins), desmosomal proteins (e.g., desmoplakin and periplakin), and cornified cell envelope proteins (e.g., cytokeratins, cornulin, and small proline-rich protein 3). Proteins belonging to the first two categories are present in most cell types including urothelial cells [39], while stratified squamous epithelium located in the urethral meatus and the vaginal tract produces proteins from all of these categories abundantly [40]. Microscopic examination of urine sediments confirmed the increased squamous epithelial cell contents with scores ≥ 2+ for most of the samples present in the four clusters (Additional file 3: Table A3), referred to as uromodulin and squamous epithelial cell clusters with vaginal bacteria (USE_V_) from here on. Uromodulin, a protein contributing to the water/electrolyte balance in the urinary tract [41], was also abundant in the samples of the USE_V_ cluster. Vaginal bacteria (*Lactobacillus* and *G. vaginalis*) were identified from 22 of 30 samples, and bacteria were absent in 5 of these samples. Proteomic data confirmed a very low level of inflammation for the USE_V_ clusters in 74% of the cases according to NAD scores as evident from the positions of the samples in the graphic of Figure 2. A representative profile of the USE_V_ cluster is LG-23, with an NAD score of 20% and low abundances of inflammation-associated proteins except for defensin-1 and S100A8 (Figure 3). Compared to the profiles for SA_112 and PM_20, all proteins contributing to inflammation were less abundant in LG_23. *G. vaginalis* can be an opportunistic pathogen in the urinary and vaginal tracts. Clustering of the host protein profiles in the USE_V_ clusters, selected for the IDs of *Lactobacillus*, *G. vaginalis*, or both species, indicated the lack of a strong immune response towards these bacterial species. We conclude that the proteomic analysis has diagnostic value by providing evidence of the absence of an infection in the urogenital tract of women.

### Urethral colonization by opportunistic pathogens

The USE_V_ clusters included three cases where common urinary tract pathogens were identified, UPEC and *K. pneumoniae*. The two cases (EC_13 and KP_55) showed low NAD and ERY scores, which was in agreement with the absence of neutrophil-triggered inflammation and vascular injury. Relative abundances of the proteins displayed in Figure 3 for these two cases and their similarity with the pattern observed for LG_23 supported the notion that immune responses characteristic for UTI were not elicited upon colonization by the bacteria. Whether the cases represent asymptomatic bacteriuria (ASB) cannot be assessed due to a paucity of knowledge on molecular-level immune responses for ASB. In summary, this data supports the notion that proteomic profiles can identify cases of urethral colonization by uropathogens without activation of the innate immune system.

### Vaginal contamination with evidence of urogenital infections

The HCL_SO_ analysis generated a UP sample cluster we called the vaginal contamination (VCO) cluster, shown in Figure 1. The VCO cluster profiles featured high abundances of cytoskeletal, desmosomal and cornified cell envelope proteins, but low or moderate uromodulin quantities, suggesting that vaginal protein content was increased in these samples. The VCO score, a quantitative ratio of five proteins expressed in cervicovaginal epithelial tissue with relatively high specificity according to the database TiGER [41] compared to uromodulin was calculated. These proteins were cornifelin, cornulin, serpin B3, galectin-7, and the proteoglycan mucin-5B. Neutrophil-specific pro-inflammatory molecules such as the protein S100-A12 and lysozyme (LYZ) and proteins indicative of or responding to vascular injury (HBA1 and fibrinogen-β, respectively) were more abundant in the VCO cluster compared to the USE_v_ cluster, as shown for KP_10 in comparison to LG_23 in Figure 3. The VCO cluster contained 14 samples, all except one derived from female patients, half of which were associated with an ID for a uropathogen. In five samples, *G. vaginalis* or *Lactobacillus* was identified. Clinical evidence suggested vaginal bleeding for the patient pertaining to sample KP_10, supporting the diagnosis of a urogenital or vaginal infection with *K. pneumoniae*. The VCO scores are included in Additional file 3: Table A3. A Wilcoxon rank sum test comparing the VCO scores of the VCO and USE_V_ clusters yielded a p-value of 0.017, suggesting that the score is useful to discern no or minor from major vaginal contamination in urine specimens. No clear assessments can be made regarding the VCO score’s usefulness to distinguish a UTI from vaginal infection.

### Proteomic analysis of urine sediments identifies microbes with sensitivity and specificity levels comparable to those of urine culture

We identified bacteria from 76 UP samples, and *Candida albicans* from one UP sample (63% of all analyzed cases). Urine cultures were performed in only 55 cases, of which 44% identified at least one pathogen, 24% commensal organisms, and 17% showed no microbial growth (Additional file 3: Table A3). In the context of pathogen identifications, proteomic data and UC results were not always in agreement (Table 1). Several reasons appear to contribute to the disagreements. The challenges of interpreting metaproteomic data based on the identified microbial proteins follow. First, protein IDs for less common uropathogens may be missed because of the absence of their protein sequences from the searched database. Bacterial species represented in the database were identified via proteomic analysis (*K. pneumoniae* and *E. faecalis*) in two cases, but the UC data suggested the presence of the phylogenetically close species *Enterobacter aerogenes* and *E. faecium*, respectively. Second, microbial organisms present in low abundance in urine are more difficult to identify in the presence of highly abundant microbes, especially if the microbial species share extensive sequence identity among orthologous proteins. The EC_85 and KP_11 profiles in (Additional file 5: Dataset A1) illustrate this problem, and in particular concerns the Enterobacteriaceae family which cause the majority of all UTIs. Inaccurate genomic annotations, e.g. missing genes, for one species (present in the UP sample) result in the identification of orthologous proteins correctly annotated in the genome of a related species (but absent in the UP sample). Small tryptic peptides are identified in a shotgun proteomic analysis, which makes incorrect protein assignments by the search algorithm more likely in cases of highly sequence identity. Third, ID of bacteria present in low counts in urine, less than ~ 10,000 cells/mL, combined with a high host proteomic background may be missed because host and microbial proteins are not separately surveyed by LC-MS/MS. Low CFU counts for bacterial organisms according to UC data showed a lower match rate with proteomic IDs than high CFU counts (Additional file 3: Table A3). In contrast, proteomic identifications of microbes have the advantages that they are culture-independent and that they provide information on virulence, antibiotic resistance, encountered stress, and growth state for the identified pathogens. Two examples revealing such comprehensive data are provided in (Additional file 5: Dataset A1). In one dataset (EC_85), proteins of UPEC, *G. vaginalis* and *Lactobacillus* were profiled. In the other dataset (KP_11), the cause of the UTI was *K. pneumoniae*. Table 1 provides an overview of the comparison of the nitrite test, identifying Enterobacteriaceae in urine based on their ability to reduce nitrates, and UC results with the proteomic data. 90% of the positive nitrite tests indeed pertained to cases of bacteriuria with UPEC or *K. pneumoniae* as the causative agent, and were invariably identified with the proteomic and UC methods as well. Unlike the proteomic analysis, nitrite tests appeared to not be sensitive enough to identify Enterobacteriaceae in ten cases. Nitrite tests were not positive in 21 cases where the ID from proteomic analyses was *G. vaginalis*. Regarding differences in pathogen IDs via proteomic versus UC methods, β-hemolytic *Streptococci* were identified by UC experiments in three cases whereas proteomic data suggested presence of *G. vaginalis*. As shown in Table 1, the numbers of matching IDs for UPEC and, to a lesser extent *K. pneumoniae*, were high. In summary, the proteomic method demonstrated higher sensitivity than the nitrite test and sensitivity and specificity levels comparable to UC. High host proteomic background in a urine sample decreases the sensitivity for microbial identification.

**Table 1:** **Proteomic identification of bacteria and comparison with urinalysis results**

**Bacterial species or genus**	**Proteomic IDs** ^**3**^	**UA: Nitrite positive** ^**4**^	**Agreement: Prot. – Nitrite** ^**6**^	**Urine culture data** ^**7**^	**Agreement: Prot. – UC data** ^**8**^
UPEC^1^	25	14	56%	17	93%
Klebsiella^2^	12	7	42%	4	75%
P. aeruginosa	3	1	33%	1	33%
Streptococcus	4	1	25%	3	0%
Staphylococcus	2	1	50%	3	33%
Enterococcus	4	1	25%	3	50%
G. vaginalis	21	0 ^5^	0%	CUG 17	43%
Lactobacillus	31	0 ^5^	0%		14%

^1^uropathogenic *Escherichia coli*; ^2^this row includes pathogens whose genomes are similar to *Klebsiella*, *Citrobacter* (1 ID for *C. koseri*), and *Enterobacter* (1 ID for *E. cloacae* and 1 ID for *E. aerogenes*); ^3^in 115 of 120 analyses, proteomic data were of sufficient quality for ID assessments; ^4^UA nitrite: nitrite analysis with a dipstick test; ^5^data excluded positive tests in cases where a uropathogen was also identified; ^6^numbers for Proteomic IDs in denominator; ^7^urine culture (UC) data were available for 55 of the 120 urine samples; commensal urogenital organisms (CUG) were identified as a group in UC tests and included *Lactobacillus* and *G. vaginalis*; ^8^agreement of proteomic and UC data pertains to percentage of matching data provided that UC data were available (Proteomic IDs in denominator).

## Discussion

Analysis of urine and its sediments is a routine component of UTI diagnostics in clinical laboratories, but suffers from poor predictive ability [42-44]. These articles cited poor negative predictive values of dipstick tests (57-76%), measuring nitrite, leukocyte esterase (LE), and hemoglobin levels. Nitrite tests are not sensitive enough and limited to the detection of the nitrate-reducing Enterobacteriaceae family. A 20-35% sensitivity range was reported [42,44]. Our proteomic data suggested 38 cases of bacteriuria caused by Enterobacteriaceae among 120 analyzed samples, and nitrite tests were positive in only 54% of the incidences. Interestingly, proteomic data also provided functional information on pathogens identified in urine sediments as it pertains to nitrate reduction, nitrite detoxification, and nitrite export activities in those UTI cases where the nitrite test was negative. In five cases with *K. pneumoniae* as the infectious agent, nitrite-inducible formate dehydrogenase, nitrite reductase, nitrite extrusion protein 1, and subunits of nitrate reductase 1 and NADPH-dependent nitrite reductase were quite abundant in the bacterial proteome. In contrast, the agreement of the results obtained from LE assays, which detect inflammation in the urinary tract and are reported to suffer from poor specificity (70-72%) [43,44], with proteomic data was good. Determining neutrophil protein content in UP samples and setting the threshold for inflammation at 30%, only 7 of 60 samples had a LE assay score of less than 2+. Again, proteomics provides functional information as it pertains to this esterase activity. Three proteolytic enzymes produced by azurophilic and specific neutrophil granules, ELANE, PTRN3, and cathepsin G, were consistently among the 50 most abundant proteins in proteomic profiles of UP samples with an LE assay score of 3+. In addition to the data we present here, there is strong evidence for the dominance of neutrophils in acute inflammatory responses following pathogen invasion of the urinary tract [14,15], recently determined to be modulated by Ly6C(+) macrophages [45]. An LE assay score of 1+ may account for poor specificity considering that 6 of 11 cases with this score revealed the presence of *Lactobacillus* and 3 cases low quantities of UPEC, suggesting colonization rather than UTI in these patients. We also compared dipstick test results for hemoglobin and proteomic data to assess red blood cell protein content to estimate the severity of hematuria in patients. We observed more than 80% agreement using as a threshold for a positive hemoglobin test ≥ 2+. In summary, the comparative evaluations of urine dipstick test components and qualitative and quantitative analyses of subsets of proteomic profiles revealed good agreement in the assessment of hematuria and inflammation, but the proteomic method was much more sensitive in the detection of Enterobacteriaceae in urine sediments.

Comparing the proteomic method with UC analysis, there was very often agreement for identifications of UPEC and *K. pneumoniae* in urine samples. This was less often observed for other pathogens, e.g. *P. aeruginosa, Enterococci* and *Streptococci* (β-hemolytic *Streptococci* as identified by UC). Proteomic analysis can discern bacteria on the species level, but only when the species from the same genus are represented in the MS search database. Among the datasets analyzed in this study, four cases pertained to *Enterococci*. Proteomic data revealed four cases of *E. faecalis*, associated with UTIs in each case considering high levels of inflammation. One case identified *E. faecium* by UC analysis; this pathogen’s protein sequences were not represented in the search database and thus attributed to *E. faecalis* by the proteomic method. The latter method identified *E. faecalis* in two cases where UC analysis reported only commensal bacteria. In one case, both methods were in agreement identifying *E. faecalis*. In contrast, β-hemolytic *Streptococci* were identified by UC with less than 10^4^ CFUs/ml in three cases, whereas proteomic data did not. Surprisingly, the latter method identified *G. vaginalis* in all three cases from the urine sediment. It is plausible that the UC methods mistook *G. vaginalis* for β-hemolytic *Streptococci*. It is also plausible that the low CFU number resulted in a lack of detection of this genus via proteomics, which detects tryptic peptides from present bacteria in a host proteomic background, thus reducing sensitivity. Furthermore, *Streptococci* have thick cell walls making it more difficult to lyse the bacteria. Lysis is required to detect the cells’ proteins via proteomic methods. Culture-based methods are lysis-independent. Overall, we conclude that UC and proteomic methods deliver results of comparable quality as it pertains to identifying pathogens which cause UTIs. Both methods require 48 hours to completion. A different MS technique, MALDI-TOF, already has impacted the way pathogens are identified from clinical samples [11,46]. The method allows very specific assignments of bacteria, but colony isolation is required first to remove the host proteome background. MALDI-TOF also does not capture immune response data and currently depends on customized databases provided by commercial entities and thus difficult to modify. In an era of rapidly advancing MS technologies and algorithms that facilitate data interpretation, we predict that shotgun proteomic methods will eventually replace the MALDI-TOF and UC methods because of culture-independent approaches, and data collection useful to evaluate the pathogen’s growth state and antibiotic resistance.

Urological pathologists want specific methods to discern UTI from ASB and vaginal contamination or vaginal infection. Using proteomics, we demonstrated that it is possible to quantify proteins enriched in cervicovaginal squamous epithelium versus proteins selectively released into urine (uromodulin). A VCO score was calculated to estimate the extent of vaginal contamination in urine samples. A high score was associated with IDs for *Lactobacillus*, *G. vaginalis* and *S. agalactiae*, commensal bacteria in the vagina and in the latter two cases opportunistic pathogens, in 75% of the 36 cases, supporting the notion that the combination of VCO scores and bacterial IDs allows an assessment of vaginal contamination of urine. Using catheter-based collection methods less prone to vaginal contamination, two microbiome studies also detected *Gardnerella* in urine [18,47]. However, there is increasing evidence of the aforementioned bacterial genera as constituents of urine microbiota. *Lactobacillus*, *Gardnerella* and *Streptococcus* were identified in 11, 9, and 5 studies, respectively, characterizing urine microbiota [38]. While we could not differentiate cases of UTI and ASB (data on clinical symptoms were unavailable), we identified three cases where low neutrophil-derived inflammation and low LE scores coincided with strong proteomic evidence of urethral colonization by a pathogen. In summary, the proteomic method may be informative as it pertains to urogenital disorders other than UTI.

The need to improve the understanding of immune responses to invading pathogens, to assess criteria of antibiotic treatment failure, and to distinguish bladder from renal infections was outlined in a review article 13 years ago [1]. The metaproteomic method we introduce here innovates the way quantitative measurements of the host immune response are performed when a pathogen or commensal organism colonizes the urinary tract. The NAD score assesses inflammation with specificity results comparable to those observed with the LE assay and microscopic counts of leukocytes. The proteomic method seems superior because more data points (35 proteins) are evaluated compared to the single data points for dipstick and microscopic tests. However, the latter methods do not require major equipment and are currently less expensive to perform. We described a set of sample clusters (NAD_1_ clusters) highly enriched for neutrophil granules proteins, including protein S100-A8, protein S100-A12, MPO, ELANE, defensin-1, LTF, and cathepsin G. The protein profiles also had increased quantities of histones which, together with MPO and ELANE, are the antibacterial effectors in neutrophil extracellular traps [48]. Neutrophil extracellular traps have not been reported for UTIs, and with the examples of *S. aureus* and *P. mirabilis* infections presented in Figure 3 this data suggests their existence.

The absence of diagnostic tests to predict ascending infections in the urinary tract and urosepsis is of considerable concern. This study included one case of suspected urosepsis caused by *P. mirabilis*. The immune response profile revealed high quantities of complement component C3, which was previously linked to augmented risk of pyelonephritis via invasion of renal tubular cells [34,49]. The pathogen also produced flagellin-1 in high abundance, an extracellular protein structure increasing bacterial swarming, according to proteomic data. Flagella-mediated motility of UPEC coincides with UTI ascending to the kidneys in a mouse model [50]. Although not investigated in depth here, both pathogen and host proteomic data may contribute to predicting the susceptibility of a patient to UTIs ascending to the kidneys. Finally, a cluster of samples for which visual examination suggested precipitation of uric acid in urine specimens was identified. The protein composition of the matrix of oxalate, struvite, and uric acid urinary stones was recently described [37]. Even though there was no evidence of stone formation in the cohort we studied, there were extensive overlaps in the proteins identified in the uric acid-enriched urine sediments and the stone matrix proteomic profiles. Further investigations are needed to assess the value of the proteomic method as it pertains to the diagnosis of ascending UTI and nephrolithiasis.

## Conclusions

We demonstrate for the first time that metaproteomic analysis of urine sediments serves multiple purposes: identifying pathogens; identifying bacteria colonizing the urinary tract; characterizing the immune response against pathogens semi-quantitatively; evaluating urine contamination from vaginal fluid. The method is a step forward for urine diagnostics in the urology, nephrology and gynecology fields and may develop into applications replacing the current non-invasive urine diagnostic pathway.

## Additional files

Additional file 1: Table A1. Metaproteomic database with 22 species for computational searches of mass spectrometry data. The file contains the list of .fasta protein databases providing UniProt taxonomy identifiers used for the searches of mass spectrometry raw data. Additional file 2: Table A2. Proteins quantified for calculation of NAD, CAC, ERY and VCO scores to assess innate immune responses, antimicrobial activities, tissue injury and vaginal contamination. The file contains a list of protein accession numbers and protein names used for the calculation of four different scores. The scores are relevant in the context of immune responses and cell/tissue specificity associated with urinary proteome data. Additional file 3: Table A3. Detailed data on urine analysis via dipstick tests, microscopy, urine cultures, and urinary pellet proteomics for 120 patients. This file contains the dipstick test results, microscopic urine analysis results, urine culture results and the proteomic data for each of the 120 human subject specimens. Additional file 4: Figure A1. Protein clusters representing distinct innate immunity pathways or distinct cell types released into urine via vascular injury or exfoliation of epithelial cells. The file contains hierarchical gene/protein clusters obtained with Pearson correlation analyses optimized for gene/protein order. Additional file 5: Dataset A1. Complete proteomic datasets from two urinary pellet samples with bacterial and human protein identifications. The file provides two examples of proteomic result files listing all protein groups identified with the Sequest^HT^ search engine and Proteome Discoverer software (Thermo Fisher).

## Abbreviations

UA: Urine analysis
UC: Urine culture
LE: Leukocyte esterase
UTI: Urinary tract infection
CAUTI: Catheter-associated UTI
UPEC: Uropathogenic *Escherichia coli*
CFU: Colony forming units
MALDI-TOF: Matrix-assisted laser desorption ionization time of flight
C3: Complement component C3
ID: Identification
UP: Urinary pellet
FDR: False discovery rate
NAD: Neutrophil activation and degranulation
CAC: Complement activation and coagulation
ERY: Erythrocyte/vascular injury
VCO: Vaginal contamination
HCL_SO_: Hierarchical clustering analysis optimized for sample leaf order
HCL_PO_: Hierarchical clustering analysis optimized for protein leaf order
