# Supplementary material for: Diagnosing inflammation and infection in the urinary system via proteomics
Source: J Transl Med. 2015 Apr 8;13:111. doi: 10.1186/s12967-015-0475-3 (PMC4396075; doi:10.1186/s12967-015-0475-3)

## Figure A1. Additional Files. Protein clusters representing distinct innate immunity pathways or distinct cell types released into urine via vascular injury or exfoliation of epithelial cells

Protein clusters representing distinct innate immunity pathways (neutrophil activation and degranulation, complement system activity and coagulation) or distinct cell types released into urine via vascular injury or exfoliation of epithelial surface cells (eryhtrocytes, squamous epithelium of vagina / urethral meatus). Protein clusters resulting from Hierarchical Clustering Analysis of urinary proteomic data from 110 samples using the Pearson correlation metric. The software tool used for the analyses was the Multiple Experiment Viewer (MeV) described in the method section. The parameters used were: selection of Gene and Sample Trees; optimized for Gene Tree Order; Absolute Distance Metric and Complete Linkage Clustering. The clusters depicted in the images below are

**Cluster A**. A cluster enriched for antibacterial and inflammatory proteins released from activated neutrophils (eosinophil cationic protein; myeloperoxidase; lactotransferrin; neutrophil defensin 1; histone H2B; Histone H3.1; cathepsin G; protein S100-A8)

**Cluster B**. A cluster enriched for immune defense proteins released from activated neutrophil granules (chitinase-3-like protein; neutrophil collagenase; neutrophil gelatinase-associated lipocalin; cathelicidin; plastin-2; cytochrom b-245 light chain; myeloblastin; protein S100-A12; protein-arginine deiminase type-4)

**Cluster C**. A cluster of proteins whose expression is enriched in stratified squamous epithelium (e.g. the vagina): cornulin, cytoskeletal 1 keratin type II, cytoskeletal 4 keratin type II, small proline-rich protein 3; small proline-rich protein 2G, cornifin-B; cellular retinoic acid-binding protein 2

**Cluster D**. A cluster of nineteen proteins derived specifically from the expression in erythrocytes

**Cluster E**. A cluster of proteins associated with the complement system and coagulation: complement components (C6, C1r, C8 alpha chain, C7, C4 beta chain, C3, C4b-binding protein, factor H, factor B, C8 gamma-chain, C8-beta chain); proteins part of or interacting with the coagulation cascade (inter-alpha-trypsin inhibitor heavy chain H1, inter-alpha-trypsin inhibitor heavy chain H1, fibronectin, prothrombin, heparin cofactor II, alpha-2-antiplasmin, coagulation factor XII, kallistatin; apolipoprotein A-II)

**Figure A1, continued.**

**Protein Cluster A**. A cluster enriched for antibacterial and inflammatory proteins released from neutrophils and eosinophils (eosinophil cationic protein; myeloperoxidase; lactotransferrin; neutrophil defensin 1; histone H2B; Histone H3.1; cathepsin G; protein S100-A8)

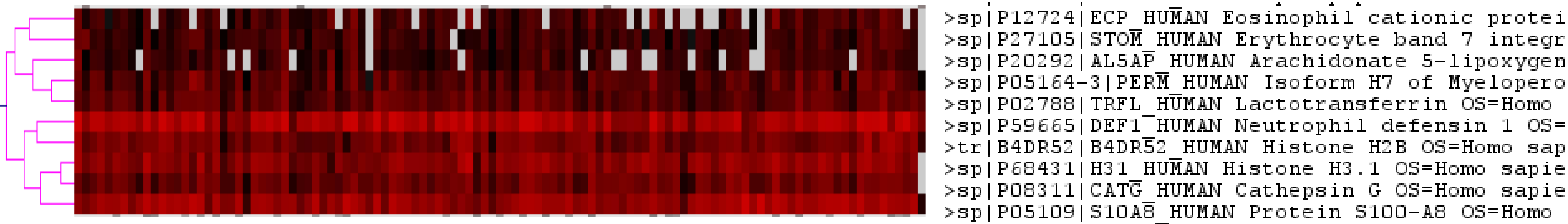


**Protein cluster B**. A cluster enriched for immune defense proteins released from neutrophil granules upon activation (chitinase-3-like protein; neutrophil collagenase; neutrophil gelatinase-associated lipocalin; cathelicidin; plastin-2; cytochrom b-245 light chain; myeloblastin; protein S100-A12; protein-arginine deiminase type-4)

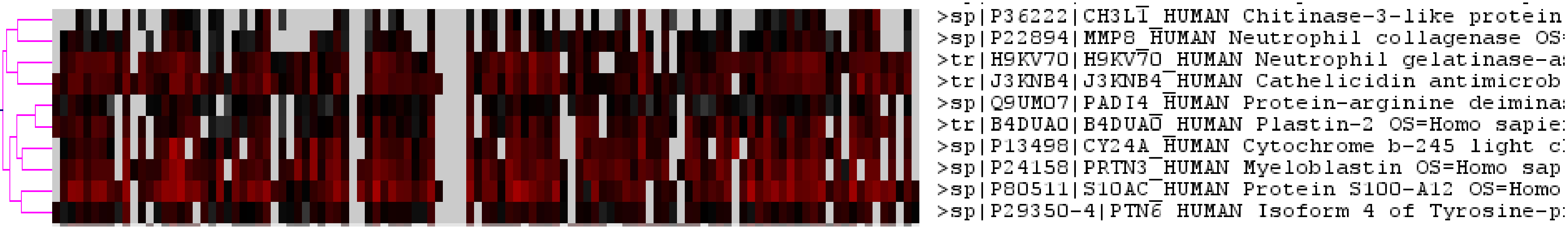


**Protein cluster C**. A cluster of proteins whose expression is enriched in stratified squamous epithelium (e.g. the vagina): cornulin, cytoskeletal 1 keratin type II, cytoskeletal 4 keratin type II, small proline-rich protein 3; small proline-rich protein 2G, cornifin-B; cellular retinoic acid-binding protein 2

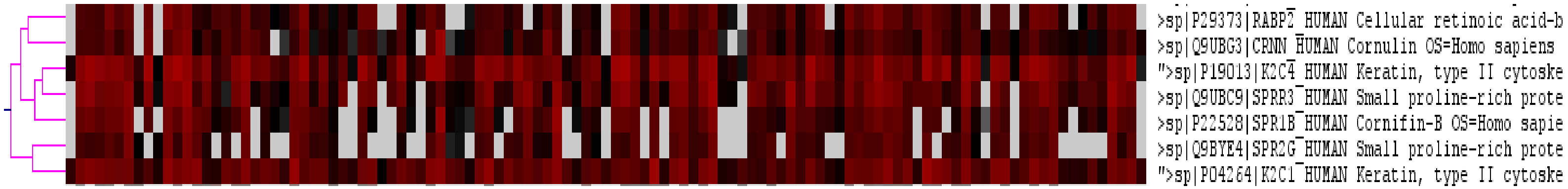

**Figure A1, continued.**

**Protein cluster D**. A cluster of nineteen proteins derived specifically from the expression in erythrocytes

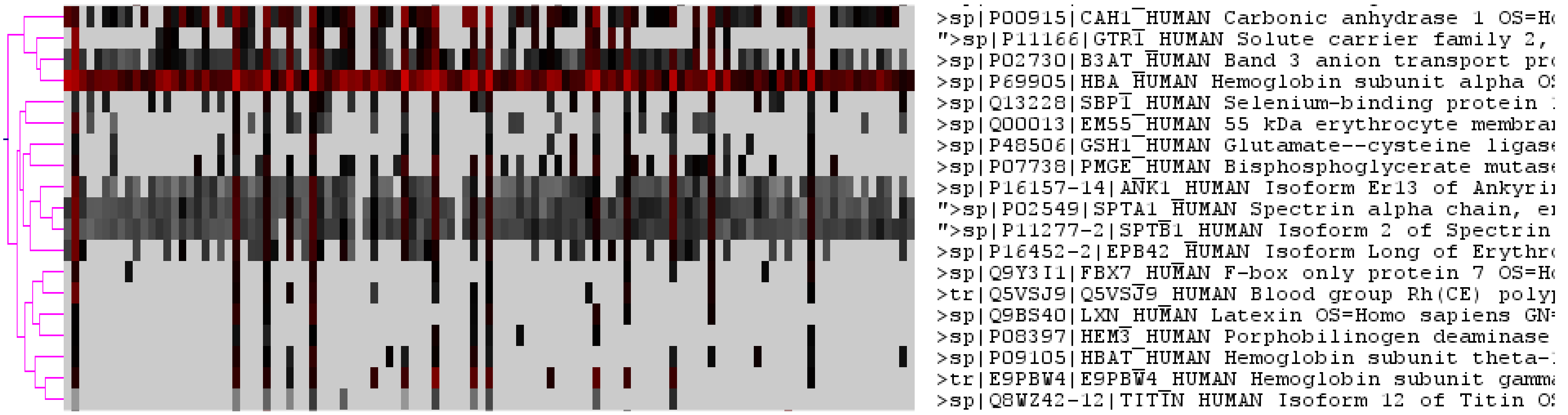


**Protein cluster E**. A cluster of proteins associated with the complement system and coagulation: complement components (C6, C1r, C8 alpha chain, C7, C4 beta chain, C3, C4b-binding protein, factor H, factor B, C8 gamma-chain, C8-beta chain); proteins part of or interacting with the coagulation cascade (inter-alpha-trypsin inhibitor heavy chain H1, inter-alpha-trypsin inhibitor heavy chain H1, fibronectin, prothrombin, heparin cofactor II, alpha-2-antiplasmin, coagulation factor XII, kallistatin; apolipoprotein A-II)

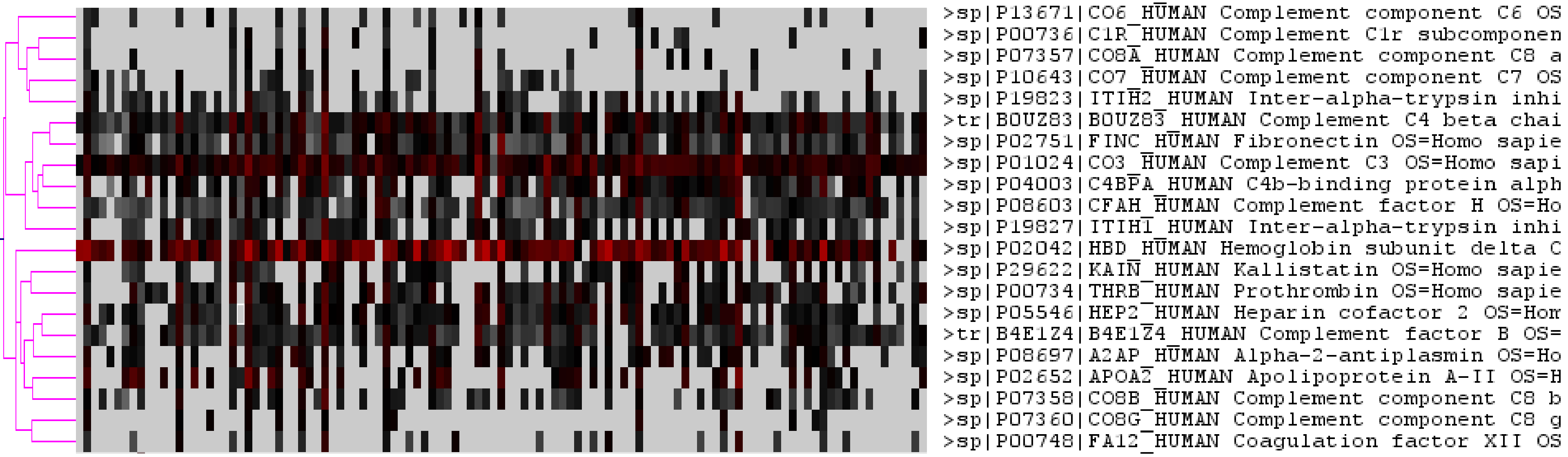

Supplement: Additional file 4: Figure A1. — Protein clusters representing distinct innate immunity pathways or distinct cell types released into urine via vascular injury or exfoliation of epithelial cells. The file contains hierarchical gene/protein clusters obtained with Pearson correlation analyses optimized for gene/protein order. [file 12967_2015_475_MOESM4_ESM.pdf]
